# Supplementary material for: Global greenhouse gas emissions from residential and commercial building materials and mitigation strategies to 2060
Source: Nat Commun. 2021 Oct 21;12:6126. doi: 10.1038/s41467-021-26212-z (PMC8531392; doi:10.1038/s41467-021-26212-z)
Supplement: Supplementary file 1 — Supplementary Information [file 41467_2021_26212_MOESM1_ESM.pdf]

Supplementary Information

Global greenhouse gas emissions from residential and commercial building materials and mitigation strategies to 2060

Xiaoyang Zhong[1*], Mingming Hu[1,2], Sebastiaan Deetman[1,3], Bernhard Steubing[1], Hai Xiang Lin[1,4], Glenn Aguilar Hernandez[1], Carina Harpprecht[1,5], Chunbo Zhang[1], Arnold Tukker[1,6], Paul Behrens[1,7*]

[1]Institute of Environmental Sciences (CML), Leiden University, 2333 CC Leiden, The Netherlands

[2]School of Management Science and Real Estate, Chongqing University, Chongqing 40045, China

[3]Copernicus Institute for Sustainable Development, Utrecht University, 3584 CB Utrecht, The Netherlands

[4]Delft Institute of Applied Mathematics, Delft University of Technology, 2628 CD Delft, The Netherlands

[5]German Aerospace Center (DLR), Institute of Networked Energy Systems, Curiestr. 4, 70563 Stuttgart, Germany

[6]Netherlands Organization for Applied Scientific Research TNO, 2595 DA The Hague, The Netherlands

[7]Leiden University College The Hague, Leiden University, 2595 DG The Hague, The Netherlands

*email: x.zhong@cml.leidenuniv.nl

*email: p.a.behrens@cml.leidenuniv.nl

Outline

1. Model framework..3

2. Region and income group classification 7

3. Scenario generation..9

4. Scenario evaluation..18

5. Additional figures and tables..21

6. Uncertainty and sensitivity analysis..22

7. Supplementary References..26

1. Model framework

The overall model framework is shown in Supplementary Figure 1. It is comprised of a building demand module, a material demand module, and a material supply module. Each module is described in turn.

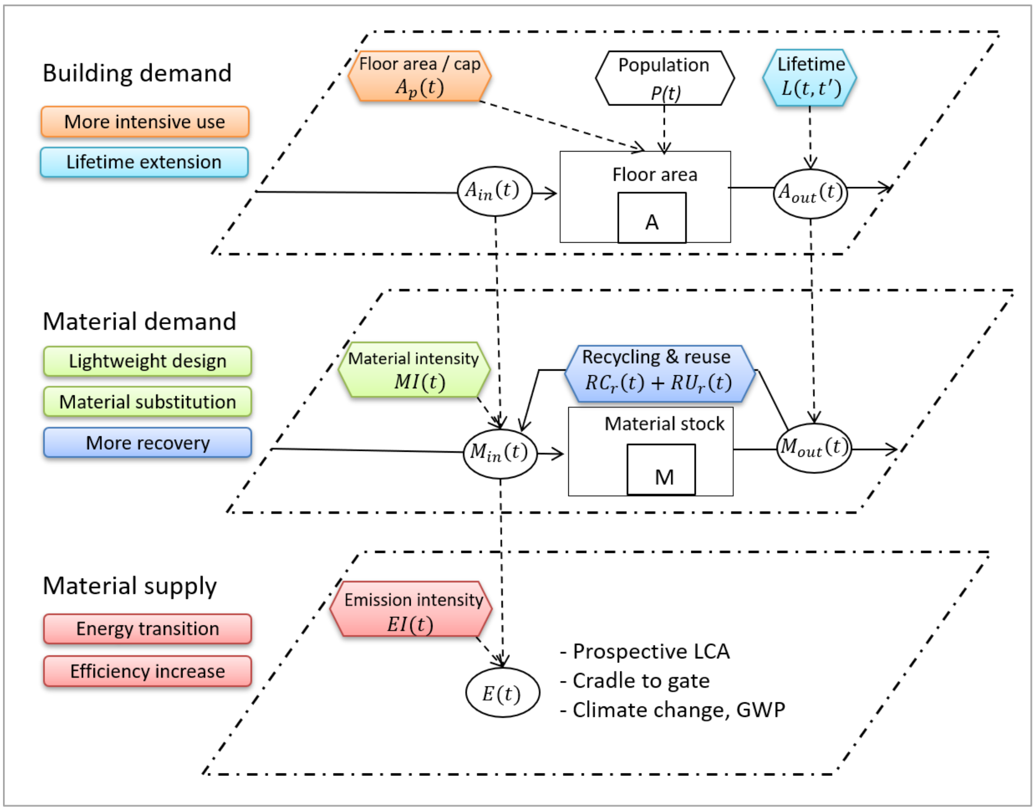

Supplementary Figure 1. The conceptual framework of the GloBUME model. This dynamic GloBUME model (global dynamic building materials use and embodied GHG emissions model) consists of three layers, i.e. a building demand layer, a material demand layer, and a material supply layer. Two rectangles in the center represent stocks of floor area and materials respectively, ovals denote flows, hexagons are the drivers or determinants. Colors indicate drivers given by each scenario, driven by related strategies illustrated in the rounded rectangles filled with the same colors. Data and codes details are freely available from the link: https://doi.org/10.5281/zenodo.5171943.

Building demand layer

In the building demand layer, the dynamic building stock and flows are calculated by the following equations:

$$A_{in}(t) = A(t) - A(t-1) + A_{out}(t) \quad (1)$$

$$A(t) = P(t) * A_p(t) \quad (2)$$

$$A_{out}(t) = \sum_{t'=t_0}^{t} A_{in}(t') * L(t,t') = \int_{t_0}^{t} A_{in}(t') * L(t,t')\, dt' \quad (3)$$

$$L(t,t') = k(t) * \lambda(t)^{-k(t)} * (t-t')^{k(t)-1} * e^{-((t-t')^{k(t)}/\lambda(t))^{k(t)}} \quad (4)$$

Equation (1) describes the fundamental balance between stocks and flows of building floor area, where $A(t)$ denotes the building demand in year t, and is the key driver of the model, determined by population, denoted by $P(t)$, and floor area per capita, denoted by $A_p(t)$, as shown in Equation (2) and Supplementary Figure 1. $A_{in}(t)$ represents the building inflow in year t and is determined by the increase in building demand, calculated by $A(t) - A(t-1)$, and the building outflow, represented by $A_{out}(t)$. The building outflow in year t, defined as the demolition of buildings which were built in different years before year t, is determined by the cohort and the building's lifetime distribution function. As shown in equation (3), t' ($t'< t$) represents the year in which the building was constructed, and $L(t,t')$ denotes the probability that the buildings built in year t' will be demolished in year t. We assume buildings' lifetime follows the Weibull distribution and use the equation (4) to calculate $L(t,t')$, where $\lambda(t)$ and $k(t)$ represent the scale parameter and the shape parameter in year t, respectively. Note that the functional form for the lifetime distributions can have a considerable impact on the calculation of outflow and inflow[1]. Other functional forms, e.g., normal[2], Log-normal[3], and Gamma functions[4] have also used in material related dynamic analysis. However, the Weibull distribution is the most common in the literature, and is therefore the distribution for which the most reliable data are available[5].

This study covers 4 types of residential buildings (detached houses, semi-detached houses, apartments, and high-rise buildings) in 2 areas (urban, and rural) and 4 types of commercial buildings (offices, retails & warehouses, hotels & restaurants, and Other commercial buildings) in 26 global regions. The data on population are derived from the integrated assessment model of IMAGE[6]. The population share by building types are derived from Marinova et. al (2019)[7]. The floor area per capita in each region differs by scenario and is discussed in the Scenario generation section (see below). The region classification is shown in Region classification section (see below).

Note that although we mainly consider social-economic factors (i.e., population and floor area per person) as the driver of the global demand in building stocks. There are other factors that will impact future building stocks and could be modelled in future work (using different modelling approaches). For example, the increasing frequency and severity of climate changes may create extra building demand as a complement for those damaged by natural disasters[8], or abandoned in areas declared uninhabitable[9].

Material demand layer

$$M_{in}(t) = A_{in}(t) * MI(\mathrm{t}) \tag{5}$$

$$M_{out}(t) = \int_{t_0}^{t} L(t,t') * A_{in}(t') * MI(t')\, dt' \tag{6}$$

Materials inflow in year t, defined as $M_{in}(t)$, is calculated by multiplying building inflow with materials use intensity per floor area in year t, denoted as $MI(t)$. We include seven major building materials, i.e. steel, concrete, brick, aluminium, copper, wood, and glass. Detailed material density data is included in the Scenario generation section (see below).

As shown in equation (6), the materials outflow in year t, denoted by $M_{out}(t)$, is determined by the cumulative sum of the floor area of demolished buildings in year t (i.e. $L(t,t') * A_{in}(t')$), and the material intensity in year t' (i.e. $MI(t')$), when the demolished buildings were built.

Material supply layer

$$E(t) = PM_{in}(t) * PEI(t) + SM_{in}(t) * SEI(t) \tag{7}$$

$$SM_{in}(t) = M_{out}(t) * RC_r(t) \tag{8}$$

$$PM_{in}(t) = M_{in}(t) - SM_{in}(t) - M_{out}(t) * RU_r(t) \tag{9}$$

Materials demand can be met by primary production, secondary production, or reuse and their emission intensities differ significantly (cradle-to-gate emission intensity is 0 for reused materials). In equation (7), $PM_{in}(t)$ and $SM_{in}(t)$ denote the quantities of materials supplied by primary and secondary production respectively, while $PEI(t)$ and $SEI(t)$ represent the GHG emission intensity of the cradle-to-gate life cycle primary and secondary material production, respectively. $PM_{in}(t)$ and $SM_{in}(t)$ can be calculated by equation (8) and (9) respectively, where $RU_r(t)$ and $RC_r(t)$ represent the reuse and

recycling rate of building materials, i.e., the percentage of post-consumer material scraps that are collected for reuse or to produce secondary materials, respectively. Note that here are some inherent assumptions: 1) we only consider the recycling and reuse of the newly generated material outflows, which means the historical scraps would not be considered for recycling and reuse in the future; 2) we only consider the recycling and reuse of material outflows within the same region, area, and building type, which means cross-regional scraps, for example, are not considered for recycling and reuse; 3) we assume that the secondary materials provide the same service as the primary materials, that is, a 1:1 substitution between primary and secondary materials in mass terms. Under these assumptions, our circularity model should be taken as a simplified approach for short-term scenario analysis where material inflows are generally larger than outflows. However, in the longer-term (i.e. beyond 2060) when material outflows largely outstrip inflows, it could be important to consider the potential of using historical scrap reserves and scraps moving across regions/areas/building types. $PEI(t)$ and $SEI(t)$ are calculated using the life cycle assessment (LCA) model based on the life cycle inventory database eco-invent, with emission intensities for unit processes specified per world region, and which we further specified for future moments in time (more information about the LCA model can be found in section Scenario evaluation in this document).

2. Region and income group classification

Supplementary Table 1 below shows the 26 world regions in this research and the countries or sub-regions included in each world region. We further classify the 26 regions into 3 income groups, i.e., high-income, upper-middle-income, and low- & lower-middle-income groups according to the World Bank Atlas classification[10] (see Supplementary Table 2). Note that we report results for the low-income and lower-middle-income regions together for ease of inspection.

Supplementary Table 1. The region classification used in the model[6]

Abbre	Region	Countries or sub-regions in the region
Canada	Canada	Canada
USA	USA	St. Pierre and Miquelon, United States
Mexico	Mexico	Mexico
C. America	Central America	Anguilla, Aruba, Bahamas, The, Barbados, Belize, Bermuda, Cayman Islands, Costa Rica, Dominica, Dominican Republic, El Salvador, Grenada, Guadeloupe, Guatemala, Haiti, Honduras, Jamaica, Martinique, Montserrat, Netherlands Antilles, Nicaragua, Panama, Puerto Rico, St. Kitts and Nevis, St. Lucia, St. Vincent and the Grenadines, Trinidad and Tobago, Turks and Caicos Isl., Virgin Isl. (Br.), Virgin Islands (U.S.)
Brazil	Brazil	Brazil
RS.America	Rest of South America	Argentina, Bolivia, Chile, Colombia, Ecuador, Falkland Islands., French Guyana, Guyana, Paraguay, Peru, Suriname, Uruguay, Venezuela, RB
N. Africa	Northern Africa	Algeria, Egypt, Arab Rep., Libya, Morocco, Tunisia, Western Sahara
W. Africa	Western Africa	Benin, Burkina Faso, Cameroon, Cape Verde, Central African Republic, Chad, Congo, Dem. Rep., Congo, Rep., Cote d'Ivoire, Equatorial Guinea, Gabon, Gambia, The, Ghana, Guinea, Guinea-Bissau, Liberia, Mali, Mauritania, Niger, Nigeria, Sao Tome and Principe, Senegal, Sierra Leone, St. Helena, Togo
E. Africa	Eastern Africa	Burundi, Comoros, Djibouti, Eritrea, Ethiopia, Kenya, Madagascar, Mauritius, Reunion, Rwanda, Seychelles, Somalia, Sudan, Uganda
S. Africa	South Africa	South Africa
W. Europe	Western Europe	Andorra, Austria, Belgium, Denmark, Faeroe Islands, Finland, France, Germany, Gibraltar, Greece, Iceland,

		Ireland, Italy, Liechtenstein, Luxembourg, Malta, Monaco, Netherlands, Norway, Portugal, San Marino, Spain, Sweden, Switzerland, United Kingdom, Vatican City State
C. Europe	Central Europe	Albania, Bosnia and Herzegovina, Bulgaria, Croatia, Cyprus, Czech Republic, Estonia, Hungary, Latvia, Lithuania, Macedonia, FYR, Poland, Romania, Serbia and Montenegro, Slovak Republic, Slovenia
Turkey	Turkey	Turkey
Ukraine	Ukraine region	Belarus, Moldova, Ukraine
C. Asia	Central Asia	Kazakhstan, Kyrgyz Republic, Tajikistan, Turkmenistan, Uzbekistan
Russia	Russia region	Armenia, Azerbaijan, Georgia, Russian Federation
M. East	Middle East	Bahrain, Iran, Islamic Rep., Iraq, Israel, Jordan, Kuwait, Lebanon, Oman, Qatar, Saudi Arabia, Syrian Arab Republic, United Arab Emirates, Yemen, Rep.
India	India	India
Korea	Korea region	Korea, Dem. Rep., Korea, Rep.
China	China region	China, Hong Kong China, Macao China, Mongolia, Taiwan
SE. Asia	Southeast Asia	Brunei, Cambodia, Lao PDR, Malaysia, Myanmar, Philippines, Singapore, Thailand, Vietnam
Indonesia	Indonesia region	East Timor, Indonesia, Papua New Guinea
Japan	Japan	Japan
Oceania	Oceania	American Samoa, Australia, Cook Isl., Fiji, French Polynesia, Kiribati, Marshall Islands, Micronesia, Fed. Sts., Nauru, New Caledonia, New Zealand, Niue, Northern Mariana Islands, Palau, Pitcairn, Samoa, Solomon Islands, Tokelau, Tonga, Tuvalu, Vanuatu, Wallis and Futuna Island
RS. Asia	Rest of South Asia	Afghanistan, Bangladesh, Bhutan, Maldives, Nepal, Pakistan, Sri Lanka
RS. Africa	Rest of Southern Africa	Angola, Botswana, Lesotho, Malawi, Mozambique, Namibia, Swaziland, Tanzania, Zambia, Zimbabwe

Supplementary Table 2. Income groups and included regions according to World Bank's classfication[11].

Income group	Regions
High-income	Canada, United States, Western Europe, Central Europe, Korea region, Japan, Oceania
Upper-middle-income	Mexico, Central America, Brazil, Rest of South America, South Africa, Turkey, Russia region, Middle East, China region, Southeastern Asia
Low-and lower-middle-income	Eastern Africa, Northern Africa, Western Africa, Ukraine region, Central Asia, India, Indonesia region, Rest of South Asia, Rest of Southern Africa

3. Scenario generation

Our model described above provides an approach of scenario explorations based on customized development trends. Note that our investigations here represent what-if scenarios rather than an accurate prediction of the future. We follow Deetman et al., 2020[5], in assuming that some variables (e.g., building lifespan, and material intensity) have remained constant in the pre-2020 period based on 2020 levels (for more details see Deetman et al., 2020[5]). For variables where historical data is available (e.g., for population, and GDP) year-specific data was used. For the future, the Baseline scenario represents a reference case where the historical trends in the building sectors around the world largely continue. The High Efficiency scenario represents a deep emission mitigation scenario where the seven strategies will be simultaneously adopted. Please see below for the full details of the strategies in each scenario.

M1-More intensive use

More intensive use represents the potential to decouple the housing demand (per capita floor area) from economic development. In the Baseline scenario, the residential floor area per capita sees increasing trends, in line with the IMAGE-SSP2 baseline scenario[6] (IMAGE data is allocated to the four building types according to Marinova et al. 2019[7]). The commercial floor area per capita towards 2050 is collected from Deetman et al. (2020)[5]and then extended to 2060 using the same regression model as that used in the original study[5]. In the High Efficiency scenario, we adopt the assumption from the literature that sees a potential for 20% reduction[12] from the Baseline values by 2050. It is noteworthy that in the High Efficiency scenario, in general, the floor area per capita will still increase (at a lower speed than in the Baseline scenario) in the rapidly urbanizing regions but slightly decrease in the highly urbanized regions. As such, the floor space gap between the rich and poorer regions will be narrowed. In future work, it may be worth exploring a more realistic and region-specific implementation of a more-intensive-use scenario, for example by adjusting the per capita floorspace differently for urban and rural areas. This would ideally account for the various drivers of floorspace demand trends (such as high demand & prices in densely populated areas) and make sure that assumptions are not at odds with achieving decent living standards or the Sustainable Development Goals[13].

M2-Lifetime extension

Short-lived buildings lead to frequent demolition of old buildings and construction of new buildings. The construction activities are associated with large amounts of building materials and GHG emissions. Therefore, prolonging service life of buildings can reduce the construction of new buildings and therefore avoid materials consumption and emissions[14, 15]. Today, the service life of buildings is fairly short and sees a potential of up to 90% extension by 2050[12]. Based on the literature[1, 5, 16], we assume that the service life of buildings follows the Weibull distribution. For residential buildings, we derive Weibull parameters (shape and scale) from Deetman et al. (2020)[5], which differ by region and the building type where possible (see details in Table S.3). We then classify the 26 regions into three groups according to the average lifetime of the buildings in each region (Supplementary Table 6). For commercial buildings, we employ a shape parameter of 1.44 and a scale parameter of 49.6 on the global average, according to the same study[5].

Under the Baseline scenario, we assume that the lifetime of buildings will stay at the current level. In the High Efficiency scenario, we assume a lifetime extension by 30%, 60%, and 90% by 2050 for residential buildings newly constructed in the regions classified as having long-, medium-, and short-lifetimes, respectively (see Supplementary Table 4). Similar assumptions are found in the literature[12], which assumed a 90% lifetime extension by 2050 in several countries. We assume a lifetime extension by 60% for newly constructed commercial buildings by 2050.

Supplementary Table 3. Weibull parameters used for modelling the lifetime of residential buildings[5]

Region	Building type	Shape	Scale
Japan	Detached	1.88	41.23
	Semi-detached	1.89	42.58
	Apartments	1.90	43.95
	High-rise	2.56	34.05
China region	Urban: Detached	2	33.85
	Urban: Semi-detached	2	39.49
	Rural: (Semi)-detached	2	31.03
	Apartments	2	53.60
	High-rise	2	56.42
Central Europe	(Semi)-detached	2.5	73.26
	Apartments	2.5	101.43
	High-rise	1.97	67.36

United States	All	4.16	85.19
Western Europe	All	2.95	70.82
Canada	All	1.97	57.53
Mexico	All	1.97	63.17
Brazil	All	1.97	112.80
Rest of South America	All	1.97	68.24
Southeastern Asia	All	1.97	56.40
Oceania	All	1.97	94.00
Global Average	All	1.97	67.34

Supplementary Table 4. Region classification according to residential building lifetime.

Classification	Regions	High Efficiency scenario
Long-lifetime	Brazil, Rest of South America, Western Europe, USA, Central Europe, Ukraine, Russia region, Oceania	30% extension by 2050
Medium-lifetime	Central America, Northern Africa, Western Africa, Eastern Africa, South Africa, Middle East, India, Korea region, Indonesia region, Rest of South Asia, Rest of Southern Africa, Turkey, Central Asia	60% extension by 2050
Short-lifetime	Japan, China region, Southeastern Asia, Canada, Mexico	90% extension by 2050

M3-Lightweight design

Today's construction practices often overuse materials due to less efficient design or a relatively low physical strength of primary materials[17, 18]. There is a potential for lightweighting through more advanced design strategies including novel structural design[19], typology optimization[20], additive construction (such as 3D printing)[21], and high strength steel and aluminium utilization[22]. For the Baseline scenario, we assume that material use intensity will stay at the current level. We used the material density of steel, concrete, copper, aluminium, glass, and wood by building type per region from Marinova et. al[7] and Deetman et. al[5]. Given that the material intensity for brick was missing in these works we used values based on multiple other peer-reviewed studies (see Supplementary Table 5). Since China is the largest brick producer (producing over 60% of the world total[23]), we also derive a specific brick dataset for China. Given the lack of data, we applied a global average for other regions. In the High Efficiency scenario, we assume that the material intensities of steel and aluminium will linearly decrease in the

future reaching a reduction of 19% [17, 18] by 2050. A reduction by 10% is assumed for concrete. The assumptions are somewhat more conservative than some national scenarios[12, 24] to account for the fact that there may be regulatory resistance and delays across the world. The intensities of other materials are assumed to remain constant.

Supplementary Table 5. The brick intensity of buildings.

Region	Building type		Brick intensity (kg per m^2)	Sources
China region	Residential	Rural	460.2	25, 26, 27
		Urban	235.6	26, 28, 29, 30, 31
	Commercial	Offices+	221.5	25, 26
		Hotels+	105	26
		Retails+	274	25, 26
		Others	339.3	25, 26, 27
Global average*	Residential	Rural	373.2	25, 26, 27, 31, 32, 33
		Urban	241.1	26, 28, 29, 30, 31, 32, 33, 34, 35, 36
	Commercial	Offices+	221.3	25, 26, 37, 38
		Hotels+	105	26
		Retails+	274	25, 26
		Others	240.9	25, 26, 31, 32, 35

Note: *In practice the regional difference in brick intensity can be considerable. The model can be improved when more data are available.

M4-Material substitution

Timber houses are generally considered an environmentally friendly substitution for steel and concrete structures for two reasons: firstly, using timber can avoid considerable emissions during material production processes[39]; secondly, timber can serve as long-term carbon storage if sources sustainably[40]. However, there are also concerns surrounding land-use change to manage forests and related biodiversity loss[41], especially in rapidly growing Asian and African regions. In the Baseline scenario, we assume no additional timber substitution compared to the current level. In the High Efficiency scenario, we assume that a 10% higher timber substitution (following a liner increase as of 2020) is achieved in new buildings by 2050. That is, a fraction (10% in 2050, for example) of new buildings are designed as wood-based while other buildings conform to the Baseline material intensity (or the light-weighted intensity when light-weight design is applied). Globally a 10% timber substitution by 2050 has been investigated as a reasonable strategy given forest sustainability and capacity to manufacture mass-timber products[42],

despite being more conservative compared to other scenarios in the literature which see 50% or even 90% scenarios[12, 42]. We derive the material intensity of the typical timber building systems from several studies[42, 43, 44] (see Supplementary Table 6). We mainly consider the change in the intensity of steel, concrete and wood. Other materials (e.g., gypsum, glass) are relatively insignificant and are therefore not considered. Similar with Churkina et al., 2020[42], we do not consider timber-based substitution for 'High-rise' buildings due to regulation and technological concerns. In future work, it may be interesting to apply different substitution strategies across regions and building types, especially when exploring more ambitious substitution scenarios[12]. Carbon storage is not considered in this study.

Supplementary Table 6. Material intensity estimates for timber-based building systems.

		Intensity estimate (kg per m^2)[42, 43, 44]		Adopted intensity in this analysis
		Regular	Wood-based	
Residential	Wood	14	187.24	187.24 (kg per m^2)
	Concrete	629.5	194	0.38 * CI_0
	Steel	84.2	1.4	0.017 * SI_0
Commercial	Wood	0	249.8	249.8 (kg per m^2)
	Concrete	608.4	-	0
	Steel	93.2	5.235	0.056 * SI_0

Note: a. The regular estimate represents an average of the buildings based mainly on steel or/and concrete. b. CI_0 and SI_0 represent the Baseline intensity (or the light-weighted intensity when lightweight-design is applied) of concrete and steel, respectively.

M5-More recovery

An increased recovery (recycling and reuse) of post-consumer scraps is considered as a material efficiency strategy. Currently, large amounts of steel, aluminium, copper, and wood are widely recycled while bulk nonmetallic materials are mostly downcycled to coarse aggregates[14]. To investigate the development of the recycling rate of different materials, we collect evidence from a series of studies. A recent study reports that the current recycling rate of steel in the construction industry is around 82%[45], This is compatible with the assumed range of 75–91% by Krausmann et al[46], who also assumed a recycling rate of 65% in 1900. Accordingly, we adopt a linear development of the steel recycling rate from 65% in (and prior to) 1900 to 82% in 2020. For copper recycling, according to the studies mentioned above, we assume a linear increase from 10%[46] in (and prior to) 1900 to 60%[45] in 2020. Globally the

building aluminium recycling rate is already high at around 85%[47]. GARC (Global Aluminium Recycling Committee) estimated the time-series evolution of the aluminium recycling rate in the building and construction sector, seeing an increase from 20% in 1950 to 89% in 2009[48]. Based on these estimates, we adopt 20% in (and prior to) 1950 to 89% in 2020, following a linear increase.

In the Baseline scenario, we assume that the adopted recycling rate of steel, copper, and aluminium in 2020 will be constant until 2060. Evidence shows that the recycling rate of steel, copper, and aluminium has a potential to reach 90%[49], 93%[45], and 95%[48] respectively. In the High Efficiency scenario, we assume a linear increase from 2020 values to the potential maximum recycling rates from the literature by 2050 and they are held constant afterwards. See a summary in TableS.7.

There is a potential for increasing the reuse of building components through increased prefabrication and modular construction design[12]. Based on case studies, we assume that up to 15%[18] of steel and concrete could be reused with these approaches. Both assumptions are somewhat conservative compared to the recent scenario analysis for several countries[12], yet were chosen considering the varying technology and regulation development patterns across global regions. In the Baseline scenario, no reuse is adopted for any material. In the High efficiency scenario, we assume a linear increase to 15% reuse for concrete and steel between 2020 and 2050 and hold constant afterwards. Note that an inherent assumption is that reused components will meet the function needed during the service life of the building. While this may be quite reasonable in our scenarios up to 2060, it may not be so for later in the century. Split lifespans of the buildings and reused components may be considered when exploring scenarios of longer time horizon, e.g., towards and beyond 2100.

Supplementary Table 7. Historical and projected maximum recycling rate of building materials.

Building material	1900	1950	2020	Maximum
Steel	65%[46]	/	82%[45]	90%[49]
Copper	10%[46]	/	60%[45]	93%[45]
Aluminium	20%[48]	20%[48]	89%[48]	95%[48]

M6-Energy transition

The trend in decarbonization of the global energy system may have significant impacts on the GHG emissions of materials production[50]. Among all energy types, the electricity system is expected to see

profound changes in the coming decades[50, 51]. We derive two global electricity mix scenarios from the Integrated Assessment Model of IMAGE[6], as developed by Mendoza Beltran et al. (2018)[51] and Cox et al. (2018)[52]. These electricity scenarios have incorporated consistent techno-socio-economic development in both energy demand (e.g., population size, income-level, and lifestyle) and energy supply (e.g., costs of competing electricity generating technologies) determinants[51]. Specifically, we apply the electricity scenarios that are compatible with SSP2-baseline and SSP2-2.6 pathways to our Baseline and High Efficiency scenarios, respectively. The SSP2-2.6 corresponds to a strong mitigation scenario aligned to the RCP2.6 (Representative Concentration Pathway), and thus realizes a radiative forcing of 2.6 W/m2 in 2100 (approximately 450 ppm CO2eq.)[53]. Biogenic carbon flows are not included in the IPCC GWP method used, which means that no credit is given to BECCS. As such, our evaluation of the emission reduction from the electricity transition may represent a lower bound. Also note that we do not take into account the transition in heat and other fuel types due to a lack of data. 2020 is set as the base year for all electricity transition scenarios. That is to say the electricity system changes apply only after 2020.

M7-Production routes and efficiency improvement

The literature indicates considerable potentials for improving the efficiency of material production processes or switching to different material production routes in the future[50, 54]. Such developments can reduce energy requirements of future material supply and thus also associated emissions[50, 54].

For the Baseline scenario, we assume the production efficiency and the production routes will not change in the future. For the High Efficiency scenario, we consider scenarios for two developments in this study. These are energy efficiency improvements and a change in market share of the primary copper production routes. First, energy efficiency increases are modeled for the production of steel, aluminium, and copper. For steel making, an improvement of 1.5% per year is assumed based on the study by Van der Voet et al. (2018)[50] who extrapolated historical data from World Steel Association[54] into the future. For aluminium production, we assume a yearly energy efficiency increase of 0.5% for aluminium smelting applying the results by Van der Voet et al. (2018)[50] who used data from World Aluminium[55]. Efficiency improvements are not considered for aluminium smelting, since historical data does not reveal specific trends in this case. For primary copper production, we assume a decrease of electricity and natural gas requirements

of 1.77% and 1.5% per year based on an exponential regression model by Harpprecht et al. (2021)[56] and Kuipers et al. (2018)[57] who apply data from Kulczycka et al. (2016)[58]. Note that 2020 is set as the base year for all energy efficiency increase scenarios and the efficiency changes apply only after 2020. For the development of production routes, we investigate the market shares of hydrometallurgical and pyrometallurgical primary copper production based on Harpprecht et al. (2021)[56] (Supplementary Table 8.).

Supplementary Table 8. Assumed market share of two primary copper production routes.

Unit: %	Primary Hydrometallurgy	Primary Pyrometallurgy
2020	17.86	82.14
2025	15.56	84.44
2030	13.56	86.44
2035	11.81	88.19
2040	10.29	89.71
2045	8.97	91.03
2050	7.82	92.18
2055	6.81	93.19
2060	5.93	94.07

Ore grade decline

There has been a secular ore grade declined over the last century for copper[59], nickel[60], zinc[61], and lead[61] due to large amounts of metal mining across the globe. A lower ore grade means that more ore needs to be processed to produce the same amount of metals[59]. This leads to increased energy requirements and higher emission intensities of primary metal supply[50]. Since copper, nickel, zinc, and lead are amongst the top seven metals consumed globally[62], their impacts play a considerable role in emission intensities of numerous production processes in the downstream supply chain[45]. To include their impacts on the building material production in the future, we consider ore grade decline scenarios and incorporate them equally into the Baseline and High Efficiency scenarios.

We derive metal-specific scenarios of future ore grades between 2020 and 2050 from Harpprecht et al. (2021)[56], which are then extrapolated into 2060 following the same trends drawn from the literature. Specifically, for copper, the ore grade scenarios are regionalized and derived from Northey et al. (2014)[59], who considered historic data on copper mining, global copper resources, various deposit types, as well

as future copper demand and supply. For nickel, zinc, and lead, our ore grade scenarios are based on metal-specific models by Van der Voet et al. (2018)[50], who derived them from historical ore grade development data[60, 61].

Time series data

For most variables, the year-to-year values are linearly interpolated between the known years and linearly extrapolated from 2050 to 2060 where applicable. However, for the change in floor area per capita driven by more intensive use, we use a cubic spline interpolation instead of linear interpolation to avoid sudden drops in the initial years, which may be caused by discontinuities in the first and second derivatives of the time-series curve[63]. To do this, we use the historical values of floor area per capita in 2010, 2015, 2018, 2019, 2020, and the assumed value in 2050 to interpolate and extrapolate unknown values (see an example in Supplementary Figure 2)

Note that recycling and reuse rates and materials reduction rates by lightweight design are assumed to be at the maximum value they can reach in each scenario by 2050 and remain unchanged after 2050.

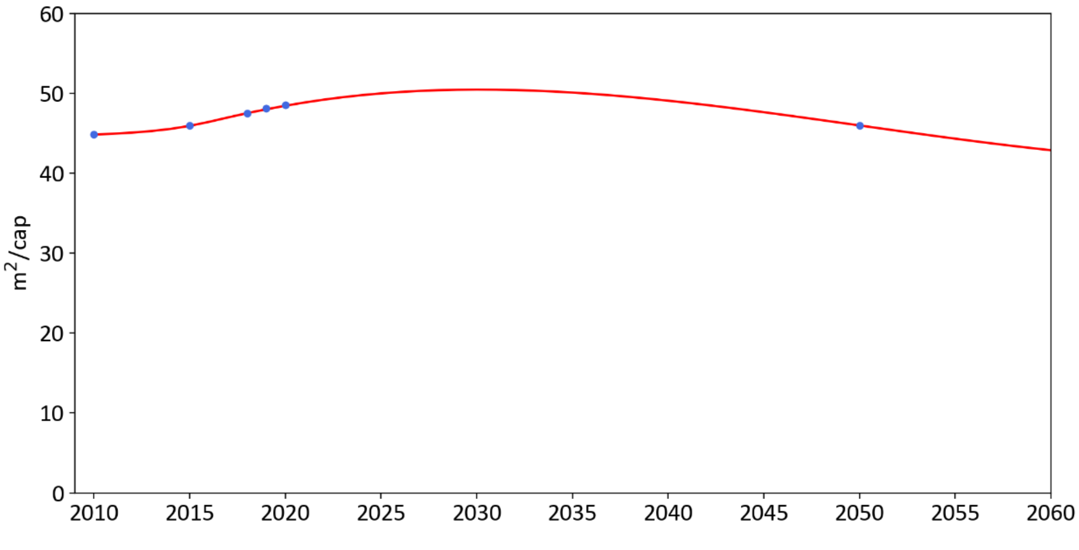

Supplementary Figure 2. An example of cubic spline interpolation for the floor area per capita in Canadian urban residential buildings during 2021-2060. This figure represents the High Efficiency scenario, where the value in 2050 sees a 20% reduction from the Baseline scenario. The blue dots represent the known values in the year 2010, 2015, 2018, 2019, 2020, and 2050.

4. Scenario evaluation

4.1 Evaluation of materials demand by scenario

Using Python, we develop a cohort-based and stock-driven dynamic building materials model to evaluate materials demand in the building sector. On this basis we assess the effect of four strategies (M1-M4, see above) on building materials demand towards 2060. A detailed introduction of the dynamic model can be found in the Model framework section above and the Methods section in the main text.

4.2 GHG emission intensity of building materials by scenario

We evaluate the GHG emission intensity of each building material using a prospective LCA model as described below. The scope is the crade-to-gate production system of per kg of each building material. We use the ecoinvent 3.6 cut-off database[64] as the life cycle inventory database because of its global coverage and regional resolution. The ecoinvent processes used are shown in Supplementary Table 9. In general, the regional mapping in ecoinvent has a different resolution with that in IMAGE. According to the approach used in Mendoza Beltran et al. (2020)[51], for ecoinvent regions that involve more than one region in IMAGE, we apply the LCA results for the larger region to all smaller regions included. For IMAGE regions involving more than one ecoinvent region, we use an average of the ecoinvent data. As an example, Supplementary Table 10 shows the regional match for the primary copper case.

For the impact category of GHG emission, we use the Global Warming Potential per substance from the IPCC 2013, with a time horizon of 100 years[65]. We use the Activity Browser (AB) software[66] to conduct the prospective LCA calculation because of its high-efficiency at scenario-based modeling. To model the development of GHG emission intensity of different unit processes in the different scenarios, we need to incorporate the relevant scenario data into the LCI database of ecoinvent. For the Baseline scenario, we incorporate the ore grade decline data into the ecoinvent database. For the High Efficiency scenario, data on ore grade decline, energy transition, efficiency improvement, and production routes development are incorporated into the ecoinvent database. To do this, we translate the scenario data of each parameter into the presamples structure[67] required for the prospective LCA calculation in the Activity Browser[66]. In practice this means that the scenario data of each parameter is specified in excel files using the approach

developed by Steubing et al. (2020)[66]. We also conduct a 'Contribution analysis' using the Activity Browser to split the contribution of CO_2 and non-CO_2 emissions to the total GHG emission.

Supplementary Table 9. The process names refer to the ecoinvent 3.6 cut-off database.

Material	Reference flow	Process
Primary steel	steel, low-alloyed	steel production, converter, low-alloyed[a]
Concrete	concrete, normal	concrete, all types to generic market for concrete, normal strength
Brick	clay brick	clay brick production
Primary aluminium	aluminium, primary, ingot	aluminium production, primary, ingot
Primary copper	copper	copper production, primary
Glass	flat glass, uncoated	flat glass production, uncoated
Wood	sawnwood, beam, hardwood, dried (u=20%), planed[b]	planing, beam, hardwood, u=20%
	sawnwood, beam, softwood, dried (u=20%), planed[b]	planing, beam, softwood, u=20%
	sawnwood, board, hardwood, dried (u=20%), planed[b]	planing, board, softwood, u=20%
Secondary steel	steel, low-alloyed	steel production, electric, low-alloyed
Secondary aluminium	aluminium, cast alloy[c]	treatment of aluminium scrap, post-consumer, prepared for recycling, at refiner
	aluminium, wrought alloy[c]	treatment of aluminium scrap, post-consumer, prepared for recycling, at remelter
Secondary copper	Copper	treatment of copper scrap by electrolytic refining

Notes: a. For simplicity, low-alloyed steel is used in consistent with ref.[50]. In practice, steel products are used in different alloys. This model can be improved when market share of alloy types is available.

b. Based on the literature review, we assume that the wood products in buildings are mainly supplied by softwood beam and hardwood (beam and board) and the ratio is 0.53:0.29:0.18[39]. The impact of the ratio assumption on the LCA result is minor because these wooden product types share similar GHGs intensities. For the secondary wood production, based on the wood cascading study, we assume that the raw wood materials are directly replaced by waste wood[68].

c. There are two major routes for post-consumer aluminium recycling: scrap remelting for wrought alloy and refining for cast alloy. Based on related studies[48, 69], we assume that 61% of scraps are to be remelted and 39% to be refined.

Supplementary Table 10. The regional mapping between IMAGE and ecoinvent for primary copper production.

Ecoinvent 3.6 region	IMAGE 3.0 region
RLA	Mexico, Central America, Brazil, Rest of South America
RNA	Canada, USA
AU	Oceania
RER	Western Europe, Central Europe, Ukraine region
RER&RAS*	Russia region
RAS	Middle East, India, Korea region, China region<br>Southeastern Asia, Indonesia region, Japan, Turkey<br>Central Asia, Middle East, Rest of South Asia
RoW	Northern Africa, Western Africa, Eastern Africa<br>South Africa, Rest of Southern Africa

Note: *For the Russia region in IMAGE 3.0, we use the average of the environmental impact intensity in RER (Europe) and RAS (Asia) from ecoinvent (for primary copper production).

5. Additional figures and tables

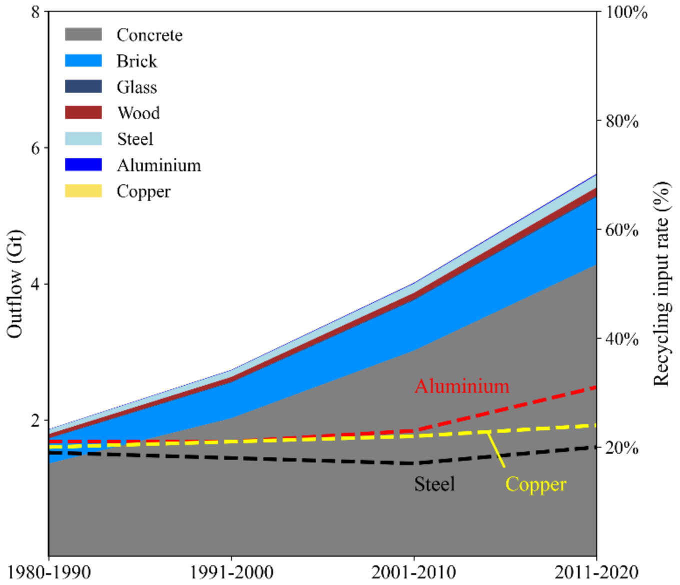

Supplementary Figure 3. Building material outflow by magnitude during 1980-2020 (left axis) and recycling input rate (the ratio of available recycled metals from outflows to annual inflows, for steel, copper and aluminium in dashed line) (right axis).

Supplementary Table 11. The eight regions classification.

Nr	Region	Sub-regions
1	E. Asia	China region, Korea region, Japan
2	N. Amer	Canada, The United States, Mexico, Central America
3	Europe	Western Europe, Central Europe, Russia region, Ukraine
4	Oceania	Oceania
5	S. Amer	Brazil, Rest of South America
6	R. Asia	Southern Asia, Indonesia region, Middle East, Central Asia, Turkey
7	S. Asia	India, Rest of Southern Asia
8	Africa	South Africa, Rest of Southern Asia, Northern Africa, East Africa, West Africa

6. Uncertainty and sensitivity analysis

The aim of this research is to evaluate long-term materials and emission trends across different regions and income groups. We then investigate the emission mitigation potentials for key material efficiency strategies. We are not able to explicitly evaluate the uncertainties across all datasets due to data limitations. However, we conduct two analyses to understand where major uncertainties may arise so as to facilitate improvements for future work.

First, we compare several primary variables in the Baseline scenario with other literature (Supplementary Table 12). For example, we employ the population trend of IMAGE-SSP2 as a representative of a middle-of-the-road path, which is comparable to the recent scenario explored by Vollset et al., (2020)[70] (with a slightly smaller estimate than the UNDP Medium variant estimate, 2019[71]). The residential floor area per capita used in this study is slightly larger than other assumptions in the literature[42, 72]. One important area of uncertainty is in commercial floor area which we derive from the time-series estimates by Deetman et al. (2020)[5], originally based on a Navigant Global Building Stock Database[73] (including service-related floor space in 231 countries in 2017). Similarly, large uncertainties are involved in the average lifetime assumptions of regional buildings. It is difficult to compare the lifetime assumptions between this study and the literature due to data limitations. In the future more detailed data along with time-series data (especially those related to the commercial buildings) are needed to improve the robustness of our analysis.

Second, we employ a sensitivity analysis to show how the High efficiency scenario is influenced by different assumptions on several variables: more intensive use, lightweight design, lifetime extension, material substitution, and more recovery. We assume a sensitivity range for each variable (see Supplementary Table 12). Note that for light weight design, increased recycling and reuse, the High efficiency scenario values are already the projected maximum from the literature. As shown in Supplementary Figure 4, emissions are sensitive to assumptions on more intensive use, whereas different assumptions on other material efficiency strategies tend not to have significant impacts on GHGs. This also indicates the importance of the consumption-based changes (leading to lower floor area per person) to emissions mitigation in the building material sector.

Supplementary Table 12. Comparison of several variables between this study and other literature.

Variable	This study	Comparable data
Population (billion)	2020: 7.67[6]<br>2060: 9.46[6]	2020: 7.79[74]<br>2060: 9.72 (8.95-10.7)[70]<br>2060: 10.15[74]
Residential space per capita (m^2/cap)	2020: 26<br>2050 Baseline: 31<br>2050 High Efficiency 25	2020: 23[72]<br>2050: 29[72]
Commercial space per capita (m^2/cap)	2020: 5.5<br>2050 Baseline: 10.29<br>2050 High Efficiency 8.24	2020: 8[72]<br>2050: 11[72]
Building lifetime	Residential: 60 (34-100)[5]<br>Commercial: 45[5]	-

Note: All data in this table are shown on a global level for the purpose of comparison.

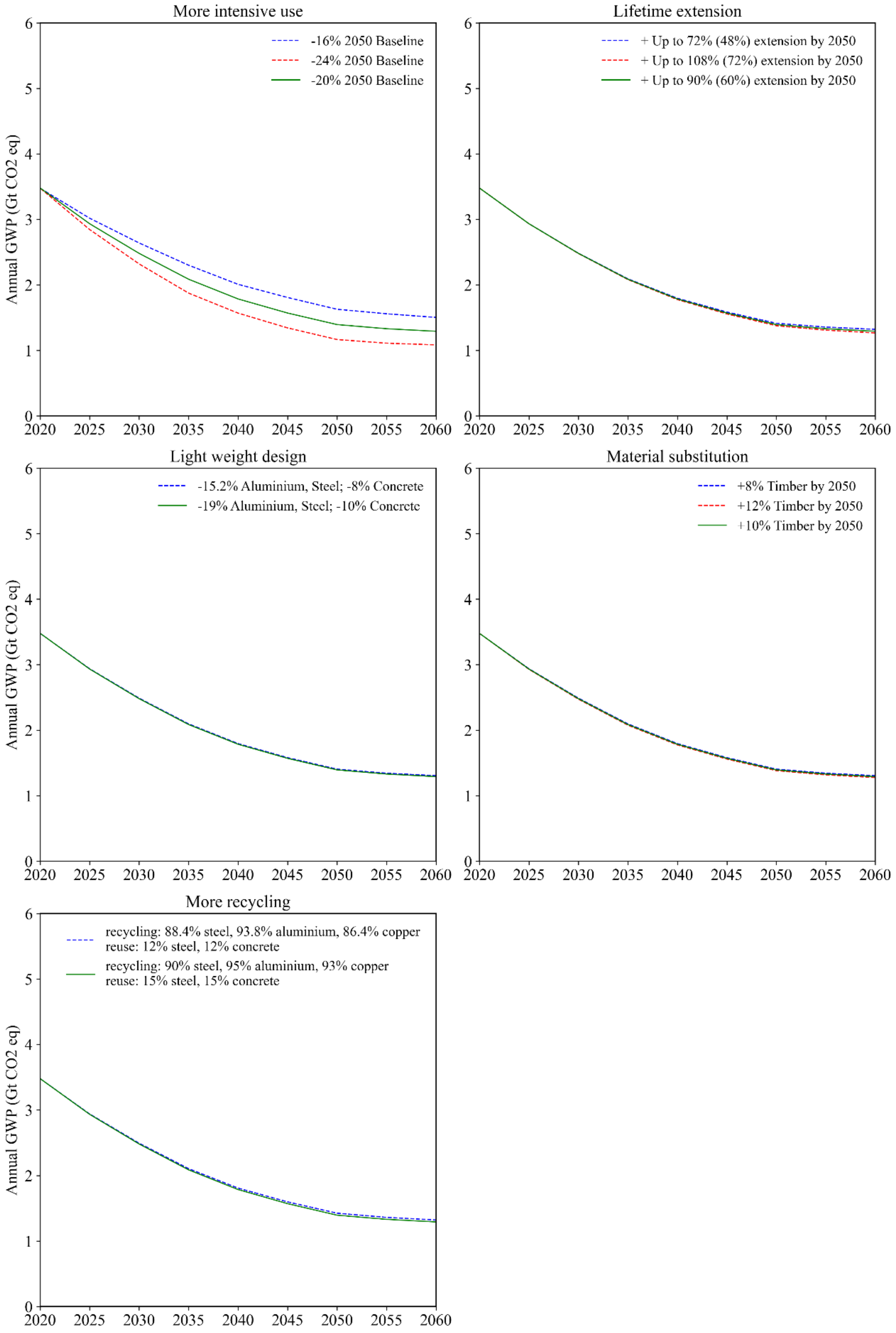

Supplementary Figure 4. The impact of different assumptions of several material efficiency

strategies on GHG emissions. The green lines in all panels represent the High efficiency scenario. More details are available in Supplementary Table 13.

Supplementary Table 13. Sensitivity ranges.

Variables	High efficiency scenario	Sensitivity range
More intensive use	20% lower area per person compared to the 2050 baseline	16%~24% lower area per person compared to the 2050 baseline
Light weight design	19% reduction in aluminium and steel, 10% in concrete by 2050	15.2%~19% reduction in aluminium and steel, 8%~12% in concrete by 2050
Lifetime extension	Lifetime extension by 30%, 60%, 90% for regions in long, medium, short lifetime groups (Supplementary Table 4)	Lifetime extension by 24%-36%, 48%-72%, 72%-108% for regions in long, medium, short lifetime groups
Material substitution	10% more timber buildings by 2050	8%~12% more timber buildings by 2050
Recycling and reuse rate	Maximum recycling rates estimated by 2050 (90% steel, 95% aluminium, 93% copper) 15% reuse of steel and concrete by 2050	88.4%~90% steel, 93.8%~95% aluminium, 86.4%~93% copper 12%~15% reuse of steel and concrete by 2050

Note: Variables not listed in this table will be consistent with the High efficiency scenario.

7. Supplementary References

1. Miatto A, Schandl H, Tanikawa H. How important are realistic building lifespan assumptions for material stock and demolition waste accounts? *Resources, Conservation and Recycling* 122, 143-154(2017).

2. Fishman T, Schandl H, Tanikawa H, Walker P, Krausmann F. Accounting for the material stock of nations. *Journal of Industrial Ecology* 18, 407-420(2014).

3. Tanikawa H, Fishman T, Okuoka K, Sugimoto K. The weight of society over time and space: A comprehensive account of the construction material stock of Japan, 1945–2010. *Journal of Industrial Ecology* 19, 778-791(2015).

4. Kapur A, Keoleian G, Kendall A, Kesler SE. Dynamic modeling of in‐use cement stocks in the United States. *Journal of Industrial Ecology* 12, 539-556(2008).

5. Deetman S, Marinova S, van der Voet E, van Vuuren DP, Edelenbosch O, Heijungs R. Modelling global material stocks and flows for residential and service sector buildings towards 2050. *Journal of Cleaner Production*, 118658(2020).

6. Stehfest E, van Vuuren D, Bouwman L, Kram T. *Integrated assessment of global environmental change with IMAGE 3.0: Model description and policy applications*. Netherlands Environmental Assessment Agency (PBL) (2014).

7. Marinova S, Deetman S, van der Voet E, Daioglou V. Global construction materials database and stock analysis of residential buildings between 1970-2050. *Journal of Cleaner Production*, 119146(2019).

8. Buchanan MK, Kulp S, Cushing L, Morello-Frosch R, Nedwick T, Strauss B. Sea level rise and coastal flooding threaten affordable housing. *Environmental Research Letters* 15, 124020(2020).

9. Xu C, Kohler TA, Lenton TM, Svenning J-C, Scheffer M. Future of the human climate niche. *Proceedings of the National Academy of Sciences* 117, 11350-11355(2020).

10. World Bank. GNI, Atlas Method (Current US $). *World Development Indicators*, (2017).

11. World Bank Group. World Bank Country and Lending Groups (Country Classification). (2020).

12. IRP. Resource Efficiency and Climate Change: Material Efficiency Strategies for a Low-Carbon Future. Hertwich, E., Lifset, R., Pauliuk, S., Heeren, N. A report of the International Resource Panel. *United Nations International Resource Panel (IRP): Nairobi, Kenya*, (2020).

13. Rao ND, Min J. Decent Living Standards: Material Prerequisites for Human Wellbeing. *Social Indicators Research* 138, 225-244(2018).

14. Hertwich EG*, et al.* Material efficiency strategies to reducing greenhouse gas emissions associated with buildings, vehicles, and electronics—a review. *Environmental Research Letters* 14, 043004(2019).

15. Wuyts W, Miatto A, Sedlitzky R, Tanikawa H. Extending or ending the life of residential buildings in Japan: A social circular economy approach to the problem of short-lived constructions. *Journal of Cleaner Production* 231, 660-670(2019).

16. Cao Z, Liu G, Duan H, Xi F, Liu G, Yang W. Unravelling the mystery of Chinese building lifetime: A calibration and verification based on dynamic material flow analysis. *Applied Energy* 238, 442-452(2019).

17. Allwood JM*, et al. Sustainable materials: with both eyes open*. Citeseer (2012).

18. Milford RL, Pauliuk S, Allwood JM, Müller DB. The Roles of Energy and Material Efficiency in Meeting Steel Industry CO2 Targets. *Environmental Science & Technology* 47, 3455-3462(2013).

19. Carruth MA, Allwood JM, Moynihan MC. The technical potential for reducing metal requirements through lightweight product design. *Resources, Conservation and Recycling* 57, 48-60(2011).

20. Bendsoe MP, Sigmund O. *Topology optimization: theory, methods, and applications*. Springer Science & Business Media (2013).

21. Ghaffar SH, Corker J, Fan M. Additive manufacturing technology and its implementation in construction as an eco-innovative solution. *Automation in Construction* 93, 1-11(2018).

22. Dhar S, Pathak M, Shukla PR. Transformation of India's steel and cement industry in a sustainable 1.5 °C world. *Energy Policy*, 111104(2019).

23. Baum E. Emissions from S Asian Brick Production & Potential Climate Impact. *Climate & Health Research Network at the Anil Agarwal Dialogue*, (2015).

24. Shanks W, Dunant C, Drewniok MP, Lupton R, Serrenho A, Allwood JM. How much cement can we do without? Lessons from cement material flows in the UK. *Resources, Conservation and Recycling* 141, 441-454(2019).

25. Huang B, Zhao F, Fishman T, Chen W-Q, Heeren N, Hertwich EG. Building Material Use and Associated Environmental Impacts in China 2000–2015. *Environmental Science & Technology* 52, 14006-14014(2018).

26. Chang Y, Huang Z, Ries RJ, Masanet E. The embodied air pollutant emissions and water footprints of buildings in China: a quantification using disaggregated input–output life cycle inventory model. *Journal of Cleaner Production* 113, 274-284(2016).

27. Huang T, Shi F, Tanikawa H, Fei J, Han J. Materials demand and environmental impact of buildings construction and demolition in China based on dynamic material flow analysis. *Resources, Conservation and Recycling* 72, 91-101(2013).

28. Cao Z, Shen L, Zhong S, Liu L, Kong H, Sun Y. A Probabilistic Dynamic Material Flow Analysis Model for Chinese Urban Housing Stock. *Journal of Industrial Ecology* 22, 377-391(2018).

29. Han J, Xiang W-N. Analysis of material stock accumulation in China's infrastructure and its regional disparity. *Sustainability Science* 8, 553-564(2013).

30. Hu D, *et al.* Input, stocks and output flows of urban residential building system in Beijing city, China from 1949 to 2008. *Resources, Conservation and Recycling* 54, 1177-1188(2010).

31. Cheng K-L, Hsu S-C, Li W-M, Ma H-W. Quantifying potential anthropogenic resources of buildings through hot spot analysis. *Resources, Conservation and Recycling* 133, 10-20(2018).

32. Marcellus‐Zamora KA, Gallagher PM, Spatari S, Tanikawa H. Estimating materials stocked by land‐use type in historic urban buildings using spatio‐temporal analytical tools. *Journal of Industrial Ecology* 20, 1025-1037(2016).

33. Surahman U, Kubota T, Higashi O. Life cycle assessment of energy and CO2 emissions for residential buildings in Jakarta and Bandung, Indonesia. *Buildings* 5, 1131-1155(2015).

34. Mesta C, Kahhat R, Santa‐Cruz S. Geospatial characterization of material stock in the residential Sector of a Latin‐American City. *Journal of Industrial Ecology* 23, 280-291(2019).

35. Tanikawa H, Hashimoto S. Urban stock over time: spatial material stock analysis using 4d-GIS. *Building Research & Information* 37, 483-502(2009).

36. Blengini GA. Life cycle of buildings, demolition and recycling potential: A case study in Turin, Italy. *Building and Environment* 44, 319-330(2009).

37. Kofoworola OF, Gheewala SH. Life cycle energy assessment of a typical office building in Thailand. *Energy and Buildings* 41, 1076-1083(2009).

38. Heeren N, Hellweg S. Tracking construction material over space and time: Prospective and geo‐referenced modeling of building stocks and construction material flows. *Journal of industrial ecology* 23, 253-267(2019).

39. Heeren N, Mutel CL, Steubing B, Ostermeyer Y, Wallbaum H, Hellweg S. Environmental Impact of Buildings—What Matters? *Environmental Science & Technology* 49, 9832-9841(2015).

40. Kalt G, Höher M, Lauk C, Schipfer F, Kranzl L. Carbon accounting of material substitution with biomass: Case studies for Austria investigated with IPCC default and alternative approaches. *Environmental Science & Policy* 64, 155-163(2016).

41. Oliver CD, Nassar NT, Lippke BR, McCarter JB. Carbon, Fossil Fuel, and Biodiversity Mitigation With Wood and Forests. *Journal of Sustainable Forestry* 33, 248-275(2014).

42. Churkina G*, et al.* Buildings as a global carbon sink. *Nature Sustainability* 3, 269-276(2020).

43. D'Amico B, Pomponi F. On mass quantities of gravity frames in building structures. *Journal of Building Engineering* 31, 101426(2020).

44. Pajchrowski G, Noskowiak A, Lewandowska A, Strykowski W. Wood as a building material in the light of environmental assessment of full life cycle of four buildings. *Construction and Building Materials* 52, 428-436(2014).

45. Elshkaki A, Graedel T, Ciacci L, Reck BK. Resource demand scenarios for the major metals. *Environmental science & technology* 52, 2491-2497(2018).

46. Krausmann F, *et al.* Global socioeconomic material stocks rise 23-fold over the 20th century and require half of annual resource use. *Proceedings of the National Academy of Sciences* 114, 1880-1885(2017).

47. Capuzzi S, Timelli G. Preparation and melting of scrap in aluminum recycling: a review. *Metals* 8, 249(2018).

48. Liu G, Bangs CE, Müller DB. Stock dynamics and emission pathways of the global aluminium cycle. *Nature Climate Change* 3, 338-342(2013).

49. Allwood JM, Cullen JM, Milford RL. Options for Achieving a 50% Cut in Industrial Carbon Emissions by 2050. *Environmental Science & Technology* 44, 1888-1894(2010).

50. Van der Voet E, Van Oers L, Verboon M, Kuipers K. Environmental Implications of Future Demand Scenarios for Metals: Methodology and Application to the Case of Seven Major Metals. *Journal of Industrial Ecology* 23, 141-155(2019).

51. Mendoza Beltran A, *et al.* When the background matters: using scenarios from integrated assessment models in prospective life cycle assessment. *Journal of Industrial Ecology* 24, 64-79(2020).

52. Cox B, Mutel CL, Bauer C, Mendoza Beltran A, van Vuuren DP. Uncertain environmental footprint of current and future battery electric vehicles. *Environmental science & technology* 52, 4989-4995(2018).

53. Riahi K, *et al.* The Shared Socioeconomic Pathways and their energy, land use, and greenhouse gas emissions implications: An overview. *Global Environmental Change* 42, 153-168(2017).

54. World Steel Association. 2016. Factsheet: Energy use in the steel industry. https://www.worldsteel.org/publications/fact-sheets.html.

55. World Aluminium. Statistics. http://www.world-aluminium.org/statistics/. Accessed December 2016.

56. Harpprecht C, van Oers, L., Northey, S. A., Yang, Y., Steubing, B. Environmental impacts of key metals' supply and low-carbon technologies are likely to decrease in the future. *Journal of Industrial Ecology*, (2021).

57. Kuipers KJJ, van Oers LFCM, Verboon M, van der Voet E. Assessing environmental implications associated with global copper demand and supply scenarios from 2010 to 2050. *Global Environmental Change* 49, 106-115(2018).

58. Kulczycka J, Lelek Ł, Lewandowska A, Wirth H, Bergesen JD. Environmental impacts of energy - efficient pyrometallurgical copper smelting technologies: The consequences of technological changes from 2010 to 2050. *Journal of Industrial Ecology* 20, 304-316(2016).

59. Northey S, Mohr S, Mudd GM, Weng Z, Giurco D. Modelling future copper ore grade decline based on a detailed assessment of copper resources and mining. *Resources, Conservation and Recycling* 83, 190-201(2014).

60. Mudd GM, Jowitt SM. A detailed assessment of global nickel resource trends and endowments. *Economic Geology* 109, 1813-1841(2014).

61. Mudd GM, Jowitt SM, Werner TT. The world's lead-zinc mineral resources: Scarcity, data, issues and opportunities. *Ore Geology Reviews* 80, 1160-1190(2017).

62. Ober JA. Mineral commodity summaries 2017. 202 (Reston, VA, 2017).

63. Fishman T*, et al.* A comprehensive set of global scenarios of housing, mobility, and material efficiency for material cycles and energy systems modeling. *Journal of Industrial Ecology* 25, 305-320(2021).

64. Wernet G, Bauer C, Steubing B, Reinhard J, Moreno-Ruiz E, Weidema B. The ecoinvent database version 3 (part I): overview and methodology. *The International Journal of Life Cycle Assessment* 21, 1218-1230(2016).

65. Qin D*, et al.* Climate change 2013: the physical science basis. *Contribution of Working Group I to the Fifth Assessment Report of the Intergovernmental Panel on Climate Change (eds TF Stocker et al)*, 5-14(2014).

66. Steubing B, de Koning D, Haas A, Mutel CL. The Activity Browser—An open source LCA software building on top of the brightway framework. *Software Impacts* 3, 100012(2020).

67. Lesage P, Mutel C, Schenker U, Margni M. Uncertainty analysis in LCA using precalculated aggregated datasets. *The International Journal of Life Cycle Assessment* 23, 2248-2265(2018).

68. Mehr J, Vadenbo C, Steubing B, Hellweg S. Environmentally optimal wood use in Switzerland—Investigating the relevance of material cascades. *Resources, Conservation and Recycling* 131, 181-191(2018).

69. Boin UMJ, Bertram M. Melting standardized aluminum scrap: A mass balance model for europe. *JOM* 57, 26-33(2005).

70. Vollset SE*, et al.* Fertility, mortality, migration, and population scenarios for 195 countries and territories from 2017 to 2100: a forecasting analysis for the Global Burden of Disease Study. *The Lancet*, (2020).

71. United Nations. World population prospects 2019: Volume II: Demographic Profiles. (2019).

72. Grubler A*, et al.* A low energy demand scenario for meeting the 1.5 C target and sustainable development goals without negative emission technologies. *Nature energy* 3, 515-527(2018).

73. T. Machinchick BF. Global Building Stock Database, Commercial and Residential Building Floor Space by Country and Building Type: 2017-2026. (2018).

74. United Nations. World population prospects 2019: Highlights. *New York (US): United Nations Department for Economic and Social Affairs*, (2019).
